# High resolution analysis of rare copy number variants in patients with autism spectrum disorder from Taiwan

**DOI:** 10.1038/s41598-017-12081-4

**Published:** 2017-09-20

**Authors:** Chia-Hsiang Chen, Hsin-I. Chen, Wei-Hsien Chien, Ling-Hui Li, Yu-Yu Wu, Yen-Nan Chiu, Wen-Che Tsai, Susan Shur-Fen Gau

**Affiliations:** 1 Department of Psychiatry, Chang Gung Memorial Hospital-Linkou, Taoyuan, Taiwan; 2 Department and Graduate Institute of Biomedical Sciences, Chang Gung University, Taoyuan, Taiwan; 3 Department of Psychiatry, National Taiwan University Hospital and College of Medicine, Taipei, Taiwan; 4 Department of Occupational Therapy, College of Medicine, Fu Jen Catholic University, New Taipei City, Taiwan; 5 Institute of Biomedical Sciences, Academia Sinica, Taipei, Taiwan; 6 Graduate Institute of Brain and Mind Sciences, National Taiwan University, Taipei, Taiwan; 7 Graduate Institute of Epidemiology and Preventive Medicine, National Taiwan University, Taipei, Taiwan

## Abstract

Rare genomic copy number variations (CNVs) (frequency <1%) contribute a part to the genetic underpinnings of autism spectrum disorders (ASD). The study aimed to understand the scope of rare CNV in Taiwanese patients with ASD. We conducted a genome-wide CNV screening of 335 ASD patients (299 males, 36 females) from Taiwan using Affymetrix Genome-Wide Human SNP Array 6.0 and compared the incidence of rare CNV with that of 1093 control subjects (525 males, 568 females). We found a significantly increased global burden of rare CNVs in the ASD group compared to the controls as a whole or when the rare CNVs were classified by the size and types of CNV. Further analysis confirmed the presence of several rare CNVs at regions strongly associated with ASD as reported in the literature in our sample. Additionally, we detected several new private pathogenic CNVs in our samples and five patients carrying two pathogenic CNVs. Our data indicate that rare genomic CNVs contribute a part to the genetic landscape of our ASD patients. These CNVs are highly heterogeneous, and the clinical interpretation of the pathogenic CNVs of ASD is not straightforward in consideration of the incomplete penetrance, varied expressivity, and individual genetic background.

## Introduction

Autism spectrum disorder (ASD) represents a group of childhood-onset neurodevelopmental disorders characterized by abnormal social interactions, impaired verbal and nonverbal communication, and the presence of restricted interests and repetitive behaviors with long-term persistence of core features and functional impairment^1,2^. The prevalence of ASD is various across different regions with an increasing trend over the years^3–5^ and with male excess in a male-to-female ratio of approximately 5:1^6–8^. In the USA, the prevalence of ASD increased in the past decade according to the report of Centers for Disease Control and Prevention of USA^8^. It was estimated that around 1 in 68 persons aged eight years in the USA in 2010 was affected with ASD^8^. However, the increasing trend of ASD prevalence was not observed in the UK^9^. The estimated prevalence of ASD in Chinese population ranged from 2.8 to 29.5 per 10,000 according to a recent review that summarized the findings in Chinese population from several areas^3^. The prevalence of ASD in Taiwan is approximately 0.3% based on the analysis of national health insurance research dataset^10^ and 1% based on the most recent Taiwan’s national survey of child and adolescent mental disorders^11^ with a male: female ratio of approximately 4: 1^10^. Due to its high prevalence, long-term impairment resulting in a great impact on individuals, families, and society^12,13^ and strong evidence of genetic components in its etiology^14^, this severe developmental disorder has been prioritized for molecular genetic studies^15^.

The heritability estimate of ASD is greater than 90%, attesting that genetic factors play a major role in the pathogenesis of ASD^16–18^. However, the genetics of ASD is very complex. Several genome-wide association studies (GWAS) have identified some common single nucleotide polymorphisms (SNPs) associated with the risk of ASD, such as common variants on 20p12.1, 5p14.1, 1p13.2^19–23^. These common SNPs, however, have only small effects on autism ASD risk, and of note, few if any of these SNPs were replicated in different studies. Furthermore, accumulating evidence suggests that rare genetic and genomic mutations also contribute to the genetics of ASD^24^. Conventional cytogenetic studies of ASD have revealed a variety of rare chromosomal abnormalities associated with ASD^25–28^ indicating aberrant genomic rearrangements are part of the genetic mechanism of ASD. Notably, the recent advent of array-based comparative genomic hybridization (aCGH) technology has discovered various submicroscopic copy number variations (CNVs) of genomic DNA associated with ASD^29,30^, leading further support to the idea that ASD is a genomic disorder in a subset of the patients. These ASD-associated CNVs are usually individually unique and of low frequency, but together they account for approximately 5–10% of idiopathic ASD^29^, hence, constituting a part of the genetic architecture of ASD^22,31,32^. The discovery of genomic mutations in ASD-associated CNVs not only helps decipher the genetic complexity of ASD^23^, but also helps shed some light on the neurobiology and pathogenesis of ASD^32–37^.

In our previous studies, we reported four pathogenic CNVs in certain ASD patients^38,39^, indicating that CNVs also play a role in the genetic architecture of ASD in our patients. To have a better understanding of the scope of rare genomic CNVs in our ASD patient population, we recruited a sample of more than 300 ASD patients and conducted a genome-wide CNV screening in this sample.

## Results

### Clinical characteristics

A total of 335 (95.7%) out of 350 cases and 1093 (98.4%) out of 1111 controls passed a series of quality control of CNV experiments. We investigated the ethnicity of cases and controls by performing principle component analysis (PCA) with SNP genotype data from all the participants of this study and the individuals included in HapMap study. The results demonstrated that the cases and controls are clustered together with the Han Chinese (Supplementary Figure 1). Therefore, the ethnicity of the participants of this study was confirmed to be the Han Chinese. Further, all the CNV data were subjected to the burden analysis. The patient group consisted of 299 boys and 36 girls with the mean age of 9.4 ± 4.0 years, while the control group consisted of 525 males and 568 females with the mean age of 68.1 ± 10.1 years. The ADI-R (Autism Diagnostic Interview-Revised) interviews revealed that the 335 patients scored 20.43 ± 6.12 in the “qualitative abnormalities in reciprocal social interaction”, 14.75 ± 4.32 in the “qualitative abnormalities in communication, verbal”, 8.19 ± 3.33 in the “qualitative abnormalities in communication, nonverbal”, and 6.95 ± 2.47 in the “restricted, repetitive and stereotyped patterns of behaviors.” All the participants with ASD were noted to have had abnormal development at or before 36 months of age. Their current average intelligence quotients (IQ) were 94.85 ± 22.55 (range, 40 to 148) for full-scale IQ, 96.74 ± 2.04 (range, 41 to 145) for performance IQ, and 95.08 ± 23.79 (range, 44 to 148) for verbal IQ. Among the 335 ASD patients, nine had been diagnosed with epilepsy (3.04%), four had been suspected of seizure (1.35%), and 19 had ever had a febrile convulsion (6.42%). These data are also provided in the Supplementary Table 1.

### CNV findings

The rates of rare CNV (<1% in the patients) at autosomes and X-chromosome were examined between the patient and control groups. CNV regions on autosomes were analyzed in all samples while CNV regions on sex chromosomes were analyzed in male samples only. We found a significant excess of the overall rate of rare CNV at autosomes in the ASD patients (2.71) compared with the control subjects (0.77). The over-representation of rare autosomal CNV rate in ASD was still present when the rare CNVs were grouped into deletion and duplication or classified according to the size as <100 kb, 100–400 kb, and >400 kb (Table 1). In the analysis of rare CNV at X-chromosome, we compared only the rate of rare CNV between male patients and male control subjects. A significant excess of the overall rate of rare CNV was observed in the male ASD patients (0.214) compared with the male control subjects (0.011). The excess rate of rare CNV at X-chromosome was still present when the CNVs were stratified into deletion/duplication, or different size groups (Table 2). We did not compare the rate of rare CNV at X-chromosome between female patients and female controls, because of the random inactivation of X-chromosome in the females. Additionally, the sample size of the female patients is small (n = 36) compared to the female controls (n = 568), and the skewed proportion of female patients vs. female controls (0.11 vs. 0.52).

**Table 1:** Comparisons of rare autosomal CNVs in patients with autism spectrum disorders and control subjects.

	CNV size	ASD (n = 335)	Controls (n = 1093)	CNV rate ASD/CON	Likelihood Ratio Chi-square	P*
Deletion	<100 Kb	333	394	0.99/0.36	109.68	<0.0001
	100–400 Kb	88	43	0.26/0.04	101.02	<0.0001
	>400 Kb	18	14	0.05/0.01	15.41	<0.0001
	Total	439	451	1.31/0.41	163.23	<0.0001
Duplication	<100 Kb	263	229	0.79/0.21	146.25	<0.0001
	100–400 Kb	150	144	0.45/0.13	84.17	<0.0001
	>400 Kb	55	19	0.16/0.02	80.21	<0.0001
	Total	468	392	1.40/0.36	224.09	<0.0001
Deletion and Duplication	<100 Kb	596	623	1.78/0.57	188.03	<0.0001
	100–400 Kb	238	187	0.71/0.17	153.20	<0.0001
	>400 Kb	73	33	0.22/0.03	89.69	<0.0001
	Total	907	843	2.71/0.77	273.33	<0.0001

P < 0.005 was considered statistically significant as it was corrected by 12 using Bonferroni test.

**Table 2:** Comparisons of rare X-chromosome CNVs in male patients with autism spectrum disorders and male controls.

	CNV size	ASD (n = 299)	Controls (n = 525)	CNV rate ASD/CON	Likelihood Ratio Chi-square	P*
Deletion	<100 Kb	10	1	0.033/0.002	14.29	0.0002
	100–400 Kb	7	1	0.023/0.002	8.98	0.0027
	>400 Kb	8	0	0.027/0	16.08	<0.0001
	Total	25	2	0.084/0.004	37.06	<0.0001
Duplication	<100 Kb	6	1	0.020/0.002	7.26	0.0070
	100–400 Kb	27	3	0.090/0.006	36.64	<0.0001
	>400 Kb	7	0	0.023/0	14.09	0.0002
	Total	40	4	0.134/0.008	55.05	<0.0001
Deletion and Duplication	<100 Kb	16	2	0.054/0.002	21.23	<0.0001
	100–400 Kb	34	4	0.114/0.008	44.93	<0.0001
	>400 Kb	15	0	0.050/0	29.94	<0.0001
	Total	65	6	0.217/0.011	88.74	<0.0001

P < 0.005 was considered statistically significant as it was corrected by 12 using Bonferroni test.

### CNVs at “hot spots”

We compared the rare CNVs found in our patients with the selected genetic “hot spots” of ASD as reported in the paper entitled “Clinical genetics evaluation in identifying the etiology of autism spectrum disorders: 2013 guideline revisions” from the practice guideline of the American College of Medical Genetics and Genomics^40^. The “hot spots” was defined as CNVs that have an especially strong association with ASD according to this paper. We identified a total of 14 patients who had pathogenic CNVs located at several of the “hot spots.” The detailed information of these CNVs including the locations, sizes, and genes encompassed in the CNV region are listed in Table 3, while the clinical data of each patient are listed in the Supplementary Table 2.

**Table 3:** CNVs at the “hot spots” identified in this study.

No.	ID	Sex	Locus	Start	End	Size (kb)	Type	Gene(s) involved	Controls (n = 1093)
1	U-1902	Male	7q31.2	116365096	116443274	78	Dup	MET	0
2	U-1638	Male	7q35	145064742	145950454	886	Dup	CNTNAP2	0
3	U-1067	Male	15q11.2-13.1	24782255	28709280	3927	Dup	PWRN1 to MIR4509 (144 gens)	1 (Dup)
4	U-1807	Male	15q11.2	23641502	28560804	4919	Dup	GOLGA6L2 to HERC2 (148 genes)	1 (Dup)
5	U-2158	Male	15q13.3	32458661	32857470	399	Del	CHRNA7	6 (1 Dup, 5 Del)
6	U-2233	Male	16p11.2	29591757	30191895	600	Dup	SMG1P2 to MAPK3 (29 genes)	0
7	U-1199	Male	22q11.21-11.22	21917141	22970127	1053	Del	UBE2L3 to LL22NC03-63E9.3 (14 genes)	0
8	U-1994	Male	22q11.21	18781534	19006984	225	Dup	LOC102725072 to DGCR9 (6 genes)	9 (Dup)
9	U-801	Male	22q11.21	18640300	21611337	2971	Dup	USP18 to FAM230B (83 genes)	0
10	U-830	Male	22q11.21	19024794	21611337	2587	Del	DGCR2,–FAM230B (70 genes)	0
11	U-1459	Male	22q13.33	51127905	51234443	107	Del	SHANK3, ACR, RABL2B, RPL23AP82	0
12	U-1957	Male	22q13.33	51087264	51234443	147	Del	SHANK3, ACR, RABL2B, RPL23AP82	0
13	U-2239	Male	22q13.32-q13.33	49388701	51188494	1800	Dup	C22orf34 to ACR (40 genes)	0
14	U-1344	Male	Xp22.31	5940647	6666470	726	Dup	NLGN4X	0*

*Only male controls (n = 525) were screened for this CNV at X chromosome.

### Other rare pathogenic CNVs

Besides the detection of CNVs at the “hot spots” in our sample, we further identified a total of 49 rare putative pathogenic CNVs according to the “American College of Medical Genetics standards and guidelines for interpretation and reporting of postnatal constitutional copy number variants”^41^ in our sample. The pathogenic CNV was defined as “documented as clinically significant in multiple peer-reviewed publications, even if penetrance and expressivity of the CNV are known to be variable. This category includes large CNVs, which may not be described in the medical literature at the size observed in the patient but which overlap a smaller interval with clearly established clinical significance”^41^. These putative pathogenic CNVs overlapped with the pathogenic CNVs reported in the Clinical Genome Resources CNVs and DECIPHER. Table 4 presents the detailed information of these CNVs including locations, types, origins, and genes encompassed. The clinical data of each patient are provided in the Supplementary Table 3.

**Table 4:** Other rare pathogenic CNVs identified in patients and control subjects in this study.

No.	ID	Sex	Locus	Start	End	Size (kb)	Type	Origin	Genes involved	Controls (n = 1093)
1	U-728	Male	1q31.1	189384373	190699301	1315	Del		BRINP3, KINC01351, LOC440704	0
2	U-1340	Male	2p13.1-12	74857355	75324605	467	Dup	Mother	MIAP, SEMA4F, HK2, LINC01291, POLE4, TACR1, MIR5000	0
3	U-866	Male	2p16.1	58164223	59360403	1196	Dup	Father	VRK2, FANCL, LINC01122	0
4	U-985	Male	2q14.3	125532806	126965458	1433	Dup	Mother	CNTNAP5	0
5	U-2170	Male	2q14.3-21.1	129497534	131211699	1714	Del	Mother	LOC101927881, to CYP4F62P (18 genes)	0
6	U-1726	Female	2q37.3	238318241	243089444	4771	Del	de novo	COL6A3 to LOC728323 (77 genes).	0
7	U-925	Male	3p24.3	19283615	19844654	561	Dup	Father	KCNH8, MIR4791	0
8	U-480	Male	4p13	44009201	44586716	578	Dup	Father	LVCAT1, KCTD8	0
9	U-2075	Male	4q12-13.1	58189742	62734625	4545	Dup	Mother	LOC101928851 to ADGRL3 (6 genes)	0
10	U-1535	Male	4q31.22	146812877	148129943	1317	Del	de novo	ZNF827 to TTC29 (8 genes)	0
11	U-1385	Male	5p12-11	45288787	46334867	1046	Dup	Father	HCN1	0
12	U-2058	Male	5p15.33	1685594	2197203	512	Dup	de novo	MIR4277 to CTD-2194D22.4 (6 genes)	1 (Del)
13	U-215	Male	5q13.2	68867282	70391241	1524	Dup	Father	GTF2H2C to LOC647859 (14 genes)	0
14	U-2075	Male	5q32	144772150	146559069	1787	Del	Father	PRELID2 to PPP2R2B-IT1 (11 genes)	0
15	U-1428	Female	7p14.1	39137061	39545773	409	Dup	Father	POU6F2, POU6F2-AS1	0
16	U-754	Male	7p22.3	1203841	1638496	435	Dup	Father	LOC101927021 to PSMG3-AS1 (8 genes)	0
17	U-890	Male	7q22.3	104,676522	105099343	423	Dup	Father	KMT2E, SRPK2, PUS7	0
18	U-717	Male	7q31.31	119313222	120243311	930	Dup	Mother	LVCAT5, KCND2	0
19	U-2829	Male	7q32.1	127266882	127670004	403	Dup	Mother	SND1, SND1-IT1, LRRC4	0
20	U-1753	Male	8p22	13277834	13841792	564	Del	Father	DLC1, C8orf48, LOC102725080	0
21	U-1130	Male	8p23.2	2179516	3890012	1710	Dup	Mother	LOC101927815, CSMD1	0
22	U-1414	Male	8p23.3	562629	1159,817	597	Dup	Mother	ERICH1, ERICH1-AS1, LOC401442	0
23	U-1753	Male	8q21.11	75793077	76234219	441	Dup	Mother	CRISPLD1, CASC9	0
24	U-363	Male	8q24.23	136620080	138711817	2092	Del	Father	KHDRBS3, LOC101927915	0
25	U-1511	Male	9p21.3	22190562	22988892	798	Dup	Father	DMRTA1, LINC01239	0
26	U-1269	Male	9q32	116314918	117370538	1056	Del	de novo	RGS3 to ATP6V1G1 (11 genes),	0
27	U-1414	Male	9q33.2-33.3	125422424	125947085	525	Dup	de novo	OR1L1 to, STRBP (16 genes)	0
28	U-1924	Male	10p11.21	34670528	35328422	658	Dup	Father	PARD3, PARD3-AS1, CUL2	0
29	U-1255	Male	10q26.2	128138653	128597833	459	Dup	Mother	C10orf90, DOCK1	0
30	U-1230	Male	12p11.1	33752330	34532722	780	Dup	Father	ALG10	1 (Dup)
31	U-1691	Male	12p11.1	33752330	34,532,722	780	Dup	Father	ALG10	1 (Dup)
32	U-1967	Male	12q24.33	130579,093	131130277	551	Dup	Mother	FZD10-AS1, FZD10, PIWIL1, RIMBP2	0
33	U-1850	Male	15q13.3	33145711	33546098	400	Dup	Mother	FMN1, TMCO5B	0
34	U-2015	Male	16p13.3	5942659	7000800	1058	Dup	Mother	RBFOX1	0
35	U-1578	Male	17p13.3	833790	1516480	683	Del	de novo	NXN to SLC43A2 (13 genes)	0
36	U-212	Male	17p13.3	172683	577890	405	Dup	Father	RPH3Al to VPS53 (6 genes)	2 (Dup)
37	U-1999	Male	17q25.3	78951153	79505624	554	Dup	de novo	CHMP6 to FSCN2 (22 genes)	0
38	U-1999	Male	17q25.3	79619226	80178991	559	Dup	de novo	PDE6G to CCDC57 (34 genes)	0
39	U-1255	Male	18p11.31-11.23	7079985	7563165	483	Dup	Father	LAMA1, LRRC30	5 (Dup)
40	U-1519	Male	18p11.32	543161	2240220	1697	Dup	Mother	CETN1 to LINC00470 (8 genes)	0
41	U-1957	Male	19q13.42-13.43	55237234	59097842	3860	Dup	de novo	KIR3DL3 to CENPBD1P1 (160 genes)	0
42	U-2131	Male	20q13.32-13.33	58022339	59007873	986	Dup	Father	PHACTR3 to MIR646 (11 genes)	0
43	U-1452	Male	22q11.1	16055171	17330096	1275	Dup	Mother	DUXAP8 to HSFY1P1 (14 genes)	0
44	U-2200	Male	Xp22.31	6467902	8135645	1668	Del		PUDP to MIR651 (7 genes)	0*
45	U-1626	Male	Xp22.31	6659217	7975015	1316	Del		PUDP to PNPLA4 (6 genes)	0*
46	U-273	Male	Xq12-13.1	67443166	67900951	458	Dup		OPHN1, YIPF6, STARD8	0*
47	U-1160	Male	Xp11.21-11.1	57384047	58438177	1054	Dup		PAAH2, ZXD8, NLRP2B, ZXDA	0*
48	U-728	Male	Xq21.33	96705629	98004847	1299	Dup		DIAPH2, DAIPH2-AS1	0*
49	U-919	Male	Xp22.31	6455151	8145721	1691	Dup		PUDP to VCX2 (7 genes)	0*

*Only male controls (n = 525) were screened for CNVs at X chromosome.

### Two-hit CNVs

**Figure 1:**
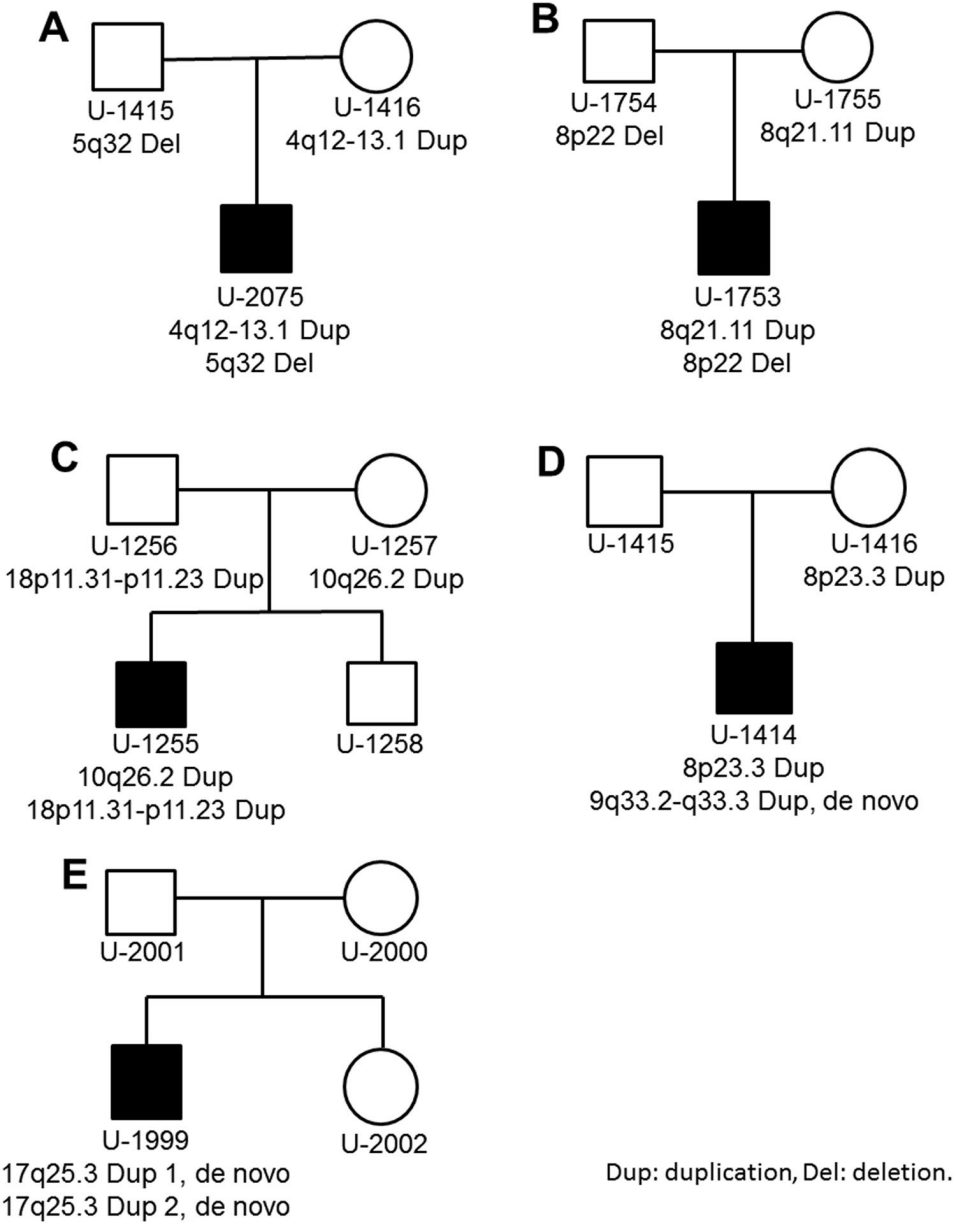
The pedigrees of five patients who carry two CNVs and the origins of these CNVs. Dup: duplication, Del: deletion.

Five patients were found to have two different putative pathogenic CNVs simultaneously in this study. Patient U-2075 inherited the 4q amplification and the 5p deletion from his mother and father, respectively (Fig. 1A). Patient U-1753 had the 8q amplification and the 8p deletion transmitted from his mother and father, respectively (Fig. 1B). Patient U-1255 acquired the 10q amplification and 18p amplification from his mother and father, respectively (Fig. 1C). Patient U-1414 had the 8p amplification from his father and a de novo 9q duplication (Fig. 1D). Patient U-1999 had two de novo amplifications at 17q25.3 simultaneously (Fig. 1E). All the parents of these five patients were carefully assessed, and none of them had ASD based on the self-administered questionnaires and clinical evaluation by the corresponding author. The detailed information of these CNVs including the locations, sizes, and genes encompassed by these CNVs are listed in Table 5, and the clinical data of each patient are provided in the Supplementary Table 4.

**Table 5:** Locations, sizes and types of CNVs in patients with two hits.

ID	Locus	Start	Stop	Size (kb)	Type	Genes contained	Controls (n = 1093)
U-2075	5q32	144772150	146559,069	1787	Del	PRELID2, GRXCR2, SH3RF2, PLAC8L1, LARS, RBM27, POU4F3, TCERG1, PPP2R2B	0
U-2075	4q12-13.1	58189742	62734625	4545	Dup	LPHN3	0
U-1753	8p22	13277834	13841792	564	Del	DLC1	0
U-1753	8q21.11	75793077	76234219	441	Dup	CRISPLD1	0
U-1255	18p11.31-p11.23	7079985	7563165	483	Dup	LAMA1	5 (Dup)
U-1255	10q26.2	128138653	128597833	459	Dup	C10orf90, DOCK1	0
U-1414	9q33.2-q33.3	125422424	125947085	525	Dup	OR1L1, OR1L3, OR1L4, OR1L6, OR5C1, OR1K1, PDCL, RC3H2, ZBTB6, ZBTB26, RABGAP1, GPR21, NCRNA00287, MIR600, STRBP	0
U-1414	8p23.3	562629	1159817	597	Dup	ERICH1	0
U-1999	17q25.3	78951153	79505624	554	Dup	CHMP6, FLJ90757, BAIAP2, AATK, LOC388428, AZI1, C170rf56, SLC38A10, C17orf55, TMEM105, BAHCC1, ACTG1, FSCN2	0
U-1999	17q25.3	79619226	80178991	560	Dup	PDE6G, C17orf90, CCDC137, ARL16, HGS, MRPL12, SLC25A10, GCGR, FAM195B, P4HB, ARHGDIA, THOC4, NANPC11, NPB, PCYT2, SIRT7, MAFG, LOC92659, PYCR1, MYADML2, NOTUM, ASPSCR1, STRA13, LRRC45, PAC3, DCXR, RFNG, GPS1, DUS1LFASN, CCDC57	0

## Discussion

In this study, we compared the frequencies of rare CNVs (<1%) between 335 patients with ASD and 1093 control subjects from Taiwan. We found a significantly higher frequency of global rare CNVs in patients with ASD compared to the control group. The significantly higher frequencies of rare CNVs in the ASD group were still present when the CNVs were subdivided into different groups based on deletion/duplication or the sizes. Our data are compatible with several previous studies^24,33,42^. Pinto and colleagues conducted a genome-wide CNV analysis of 996 ASD individuals of European ancestry and 1,287 matched controls. They found a higher global burden of rare genic CNVs in ASD patients^43^. The findings were replicated by the same group in another genome-wide CNV analysis consisted of 2,446 families with ASD^33^. In our study, we did not limit our CNV analysis to genic CNVs only, as non-genic CNVs may have position effect to affect the expression of genes outside the CNV regions. Our findings of increased global burden of rare CNVs in ASD indicate that genomic rearrangement is one of the genetic mechanisms of ASD.

Some other studies reported increased burden of CNV in female patients. Jacquemont and colleagues reported that in a sample of 762 ASD families, they found a 3-fold increase in deleterious autosomal CNVs in female patients compared to male probands^44^. Desachy and colleagues recently reported that mothers of patients with autism had a higher deletion burden than control mothers in a matched case–control population. Also, to their surprise, they found a higher autosomal burden of large, rare CNVs in females in the population. They speculated that the increased rare CNV burden in females in general population might contribute to the decreased female fetal loss in the population, but the ASD-specific maternal CNV burden may contribute to high sibling recurrence^45^. In our study, we did not conduct the similar analysis because of the relatively small sample size of female patients.

In our CNV analysis, we identified 14 patients who had CNVs located at the ASD “hot spots” as reported in the “Clinical genetics evaluation in identifying the etiology of autism spectrum disorders: 2013 guideline revisions”^40^. Our findings support the strong association of the CNVs at “hot spots” with ASD in our patient population. Among these CNVs, CNVs located at 22q11.2, 22q13.3, and 15q11-13 are the most common in our sample. Besides the CNVs located at the “hot spots,” we also detected 49 rare (<1%) CNVs larger than 400 kb that overlapped with the pathogenic CNVs reported in the Clinical Genome Resources and DECIPHER. These CNVs met the criteria of “pathogenic” according to the “American College of Medical Genetics standards and guidelines for interpretation and reporting of postnatal constitutional copy number variants”^41^. Some of these rare putative pathogenic CNVs were inherited, while some were de novo mutations. The broad distributions of both “hot spots” CNVs and rare pathogenic CNVs detected in our patients suggest extremely high genetic heterogeneity of ASD in our patients. Some studies suggested that female patients have a higher burden of CNV than male patients^33,44,45^. But, in our study, all the patients who had CNVs at “hot spots” were male (Table 3), and there were only two female patients out of 49 who had other rare (<1%) pathogenic CNVs (Table 4). The discrepancy might be due to the disproportion of female patients in this study (36 females vs. 299 males).

In this study, five patients were found to have the concomitant presence of two rare pathogenic CNVs in their genome. The finding is consistent with our previous report of a patient who had inherited two CNVs from his parents and supported the two-hit model of ASD^39^. Several studies also proposed the idea of two-hit and the multiple-hit models of ASD, suggesting that genetic underpinnings of ASD stem from combinatorial effects of mutations of oligogenic or multiple genes in different loci^39,46–48^. Leblond and colleagues reported three patients with deletions at *SHANK2* gene locus. Also, these three patients had another inherited CNV at 15q11-13 that was associated with other psychiatric disorders^46^. Two patients carried a duplication of nicotinic receptor *CHRNA7*, and one patient had a deletion of the synaptic translation repressor *CYFIP1* ^46^. Stenberg and Webber conducted a pathway-association test of target genes regulated by fragile-X mental retardation protein (FMRP) in ASD patients; they found rigorous support for the multiple-hit genetic etiology of ASD^47^. In fact, emerging evidence suggests the presence of multiple pathogenic CNVs in psychiatric patients is not rare. Hu and colleagues recently reported a novel maternally inherited 8q24.3 and a rare paternally inherited 14q23.3 CNVs in a family with neurodevelopmental disorders^49^. Williams and colleagues conducted CNV analysis in patients with velo-cardio-facial syndrome (VCFS), regardless of having psychosis or not. They found a significantly higher proportion of second CNV hit in patients with psychosis, suggesting the two-hit hypothesis may be relevant to a proportion of VCFS patients with psychosis^50^. Rudd and colleagues found a slightly higher proportion of multiple conservative CNVs in schizophrenia patients compared to controls, indicating a potential role for a multiple-hit model in schizophrenia^51^. Hence, it is likely that the multiple-hit (including two-hit) might be a commonly important genetic mechanism associated with ASD. In this study, we reported 5 patients who had two putative pathogenic CNVs larger than 400 kb. We believe that if CNVs smaller than 400 Kb were included for analysis in the future, we might find more patients with two-hit and multiple-hit of CNVs. In the family study of these 5 patients with two-hit CNVs, we found that some of these putative pathogenic CNVs were inherited from their parents, and some were a de novo mutation. However, the parents who carried one putative pathogenic CNV did not manifest ASD symptoms after careful clinical evaluation, suggesting the incomplete penetrance of these inherited pathogenic CNVs. Further, we searched for these CNVs in 1093 control subjects, and found none of these CNVs in the control group, except the duplication of 18p11.31-p11.2 (483 kb), which was found in 5 out of 1093 control subjects (0.46%). These data provided further evidence to support that these CNVs may confer increased risk to ASD. In the future, we might be able to identify unaffected carriers of high-risk CNVs if more data are accumulated.

The identification of pathogenic CNVs associated with ASD may help discover candidate genes of ASD. The genes encompassed by the CNVs in our patients are listed in Tables 3,4 and 5. Notably, several genes had been reported to be associated with autism or the other major psychiatric disorders, such as *TACR1* ^52^, *CNTNAP5* ^53,54^, *ADGRL3* ^55^, *ZNF827* ^56^, *POU6F2* ^20^, *KMT2E* ^57^, *KCND2* ^58^, *SND1* ^59^, *CNTNAP2* ^60,61^, *CSMD1* ^62,63^, *PARD3* ^64^, *HERC2* ^65^, *FMN1* ^66^, and *RBFOX1* ^67^. These findings not only provide further clues to indicate the highly genetic heterogeneity of ASD but also indicate the pleiotropic clinical effects of the mutation of these genes. Accumulating evidence showed that shared heritability and genetic mutations among different categories of psychiatric disorders seem to be regular rather than exceptional. A study of analyzing the genotype data from the Psychiatric Genomics Consortium (PGC) for cases and controls in schizophrenia, bipolar disorder, major depressive disorder, ASD and attention-deficit/hyperactivity disorder (ADHD) revealed moderate to high shared genetic etiology of these psychiatric disorders^68^. Li and colleagues recently reported that the prevalence of de novo mutations shared by four different categories of neuropsychiatric disorders: autism spectrum disorder, epileptic encephalopathy, intellectual disability, and schizophrenia, was significantly elevated^69^.

The present study has several limitations. First, to the best of our knowledge, our study has the largest sample size of Chinese population compared to the other studies^70,71^. However, the relatively limited sample size of this study is an apparent limitation to have a more comprehensive picture of CNVs in our patient population, especially in consideration of the high heterogeneity of CNVs associated with ASD. Second, in this study, we only searched for pathogenic CNVs larger than 400 kb because larger CNVs are more likely to be pathogenic^72^. We understand that small CNVs can be pathogenic. Hence, further analysis of the CNVs smaller than 400 kb will discover more ASD-associated CNVs in our patients. Third, the phenotypical interpretation of pathogenic CNVs found in our study is not straightforward given the incomplete penetrance, varied expressivity and pleiotropic effects of pathogenic CNVs identified in our sample. Also, we cannot exclude the interaction of these CNVs with the genetic background and other yet to be identified genetic or genomic mutations in the affected patients.

In conclusion, we found a significantly increased global burden of rare CNVs in our ASD patients compared to the control subjects, indicating that rare CNVs play a part in the genetic landscape of ASD in our population. Also, we identified several pathogenic CNVs at “hot spots” and various private putative pathogenic CNVs in our patients, suggesting high genetic heterogeneity of ASD in our patients. Our study also supports that high-resolution oligonucleotide SNP array is a useful tool to uncover the genetic underpinnings of patients with ASD. In the future, we will continue to analyze our data with the size of CNV smaller than <400 kb, and we expect to find more pathogenic CNVs associated with ASD, more candidate genes of ASD, and more patients with double-hit of CNV. Thus, we will have a better understanding of the genetic architecture of ASD in our population.

## Materials and Methods

### Participants and Procedures

The study protocol was approved by the Research Ethics Committee at National Taiwan University Hospital (approval number: 9561709027), Taipei, Taiwan, and Chang Gung Memorial Hospital-Linkou (approval number: 93-6244), Taiwan, for the recruitment of the patients with ASD, and Academia Sinica (approval number, AS-IBMS-MREC-91-10), Taiwan for the control group. All the experiments and informed consent procedures were performed in accordance with relevant guidelines and regulations set by the research ethics committees of the three institutes.

### Patient Participants with ASD

The study was part of the molecular genetics study of patients with ASD who were Han Chinese residing in Taiwan. The detailed recruitment and evaluation of the patients and their family members were described in our previous publication^73^. In brief, patients aged 3 to 17 years old and met the clinical diagnosis of autistic disorder as defined by the Diagnostic and Statistical Manual of Mental Disorders-IV (DSM-IV)^74^ were recruited from the Children’s Mental Health Center, National Taiwan University Hospital, Taipei, Taiwan and Department of Psychiatry, Chang-Gung Memorial Hospital, Kuei-Shan, Taiwan. The clinical diagnoses were made by board-certified child psychiatrists experienced in the assessment and intervention for ASD and were further confirmed by interviewing the parents using the Chinese version of the Autism Diagnostic Interview-Revised (ADI-R)^75,76^. The ADI-R, translated into Mandarin by Gau and colleagues, was approved by Western Psychological Services in 2007 as the ADI-R in the Chinese language^75,77^. All these patient participants further received clinical evaluation according to the DSM-5 diagnostic criteria for ASD^1^, which revealed that all the 350 participants with DSM-IV autistic disorder met the diagnosis of DSM-5 ASD. Moreover, all these patient participants received intelligence tests. For ages of 3 to 7.5 years, the Wechsler Primary and Preschool Scale of Intelligence-Revised (WPPSI-R) was given; for ages of 6 to 16 years 11 months, the Wechsler Intelligence Scale for Children-3^rd^ Edition (WISC-III) was given; for ages of 16 years and above, Wechsler Adult Intelligence Scale (WAIS) was given.

Patients with known chromosomal abnormalities and associated medical conditions including fragile X syndrome and Rett’s disorder based on DNA testing or clinical assessments were not included during the recruitment process^28,78^. Also, probands with previously identified chromosomal structural abnormality associated with autism or had any other major neurological or medical conditions were also excluded^28^. The study protocol was approved by the Research Ethics Committee of National Taiwan University Hospital (approval number, 9561709027) and Chang Gung Memorial Hospital (approval number, 93-6244), Taiwan. Written informed consents were obtained from the participants (if applicable, otherwise, child assent) and their parents after the procedures were fully explained. Genomic DNA was prepared from peripheral blood of each participant using Gentra Puregene Blood kit according to the manufacturer’s instructions (Qiagen, Hilden, Germany).

### Healthy control subjects

The control subjects (n = 1111) were chosen from the Han Chinese Cell and Genome Bank (HCCGB) in Taiwan who received physical check-up and questionnaire screening to ensure that they did not have any abnormal physical condition and mental illness^79^. Written informed consents were obtained from the participants after the procedures were fully explained.

### CNV analysis

We used Affymetrix Genome-Wide Human SNP Array 6.0 (Affymetrix, Santa Clara, CA, USA) for genome-wide CNV screening. The SNP 6.0 array contains more than 1.8 million markers including more than 906,600 probes for SNPs and more than 946,000 probes for CNVs. These probes are evenly distributed across the whole genome with a median distance between probes of ~0.7 kb. The microarray experiment was conducted by the National Genotyping Center (Academia Sinica, Taipei, Taiwan) (http://ncgm.sinica.edu.tw/ncgm_02/index.html). The hybridization intensities were captured by GeneChip Scanner 3000 (Affymetrix, Santa Clara, CA). CNVs were called using Affymetrix Genotyping Console software v.4.1 (Affymetrix, Santa Clara, CA). The average call rate was 99.49 ± 0.29%, and all samples passed genotyping quality control (call rate >= 95%). The gender was called based on the cn-probe-chrXY-ratio_gender method from Affymetrix Power Tools (Affymetrix, CA, USA). Samples with mismatched gender between computed gender and case information were excluded from analysis. Duplicated samples detected by Kinship analysis using P-Link software were also excluded. Twenty contiguous deletion or duplication probe signals were called in this study. CNV regions overlapped with centromeric regions (hg19, UCSC), antibody variable regions (PennCNV, http://www.openbioinformatics.org/penncnv/penncnv_faq.html#ig) and T-cell receptor loci (NCBI Gene, http://www.ncbi.nlm.nih.gov/gene/) were filtered out. The CNVs with the size equal or larger than 10 Kb and with the frequency of less than 1% in the patients were selected for analysis in this study. Copy number variations were considered to localize at the same locus if they overlapped by at least 80% of their length. Genes overlapped with the CNV regions were reported according to UCSC genes (NCBI37/hg19). The ethnicity of cases and controls was assessed by performing principle component analysis (PCA) with SNP genotype data from all the participants of this study and the individuals included in HapMap study.

### Burden assay

Both genic and non-genic CNVs were included for analysis. Likelihood Ratio Chi-square test was used to compare the difference of CNV rate between ASD and healthy controls with a pre-selected alpha value at P value less than 0.05. Bonferroni correction was used to adjust for the multiple testing. Thus, the significance level of the p-value was set at 0.005.

### Real-time quantitative PCR (RT-qPCR)

RT-qPCR was used to validate the CNVs detected in this study and for a family study to identify their parental origin. RT-qPCR was performed using the SYBR-Green PCR reagents kit (Applied Biosystems, Forster City, California, USA), and the CNV was assessed using a relatively standard method in the laboratory^38^. The experiment was implemented using the ABI StepOnePlus following the manufacturer’s protocol (Applied Biosystems, Forster City, California, USA). The description of primer sequences, optimal annealing temperature, and the amplicon sizes is available upon request.

### Pathogenic CNV evaluation

The pathogenic CNVs were evaluated according to two practice guidelines from the American College of Genetics and Genomics. First, according to the “Clinical genetics evaluation in identifying the etiology of autism spectrum disorders: 2013 guideline revisions”^40^, our CNVs results overlapped with those at the “hot spots” reported in this guideline are considered as pathogenic. Second, for rare CNVs outside the “hot spots,” we focused on the analysis of rare CNVs equal or larger than 400 kb for convenience’s sake. Although large CNVs are more likely to have clinical significance, we understand that small CNV can be pathogenic, and large CNV can be benign. CNVs overlapped with the pathogenic CNVs reported in the Clinical Genome Resources (https://www.clinicalgenome.org/), or DECIPHER (https://decipher.sanger.ac.uk/) were defined as pathogenic according to the “American College of Medical Genetics standards and guidelines for interpretation and reporting of postnatal constitutional copy number variants”^41^. The sizes of CNVs and genes encompassed by the CNVs were generated according to the gene annotation of the UCSC genome browser (GRCh37/hg 19) (http://genome.ucsc.edu/cgi-bin/hgGateway).

## Electronic supplementary material

Supplementary information
