# Supplementary material for: High resolution analysis of rare copy number variants in patients with autism spectrum disorder from Taiwan
Source: Sci Rep. 2017 Sep 20;7:11919. doi: 10.1038/s41598-017-12081-4 (PMC5607249; doi:10.1038/s41598-017-12081-4)
Supplement: Supplementary file 1 — Supplementary information [file 41598_2017_12081_MOESM1_ESM.doc]

**High resolution analysis of rare copy number variants in patients with autism spectrum disorder from Taiwan**

Chia-Hsiang Chen^1,2^, Hsin-I Chen^3^, Wei-Hsien Chien^4^, Ling-Hui Li^5^, Yu-Yu Wu^1^, Yen-Nan Chiu^3^, Wen-Che Tsai^3^, Susan Shur-Fen Gau^3,6,7^,*

^1^Department of Psychiatry, Chang Gung Memorial Hospital-Linkou, Taoyuan, Taiwan. ^2^Department and Graduate Institute of Biomedical Sciences, Chang Gung University, Taoyuan, Taiwan. ^3^Department of Psychiatry, National Taiwan University Hospital and College of Medicine, Taipei, Taiwan. ^4^Department of Occupational Therapy, College of Medicine, Fu Jen Catholic University, New Taipei City, Taiwan. ^5^Institute of Biomedical Sciences, Academia Sinica, Taipei, Taiwan. ^6^Graduate Institute of Brain and Mind Sciences. ^7^Graduate Institute of Epidemiology and Preventive Medicine, National Taiwan University, Taipei, Taiwan

Running title: CNV of ASD in Taiwan

Word count: abstract (197), full text (4162), 5 tables, 1 figure, 80 references, 4 supplementary tables.

*Corresponding author

Susan Shur-Fen Gau, M.D., Ph.D.

Department of Psychiatry, National Taiwan University Hospital & College of Medicine

No. 7, Chung-Shan South Road, Taipei 10002, Taiwan.

Tel : +886-2-23123456 ext. 66802; Fax: +886-2-23812408

E-mail: [gaushufe@ntu.edu.tw](mailto:gaushufe@ccms.ntu.edu.tw)

**Supplementary Figure 1. The ethnicity of cases and controls by performing principle component analysis (PCA) with SNP genotype data from all the participants of this study and the individuals included in HapMap study**

**ASW** African ancestry in Southwest USA

**CEU** Utah residents with Northern and Western European ancestry from the CEPH collection

**CHB** Han Chinese in Beijing, China

**CHD** Chinese in Metropolitan Denver, Colorado

**GIH** Gujarati Indians in Houston, Texas

**JPT** Japanese in Tokyo, Japan

**LWK** Luhya in Webuye, Kenya

**MXL** Mexican ancestry in Los Angeles, California

**MKK** Maasai in Kinyawa, Kenya

**TSI** Toscani in Italia

**YRI** Yoruba in Ibadan, Nigeria

**Supplementary Table 1. The clinical data of all 335 ASD patients participated in this study.** Of the 335 cases, 299 are male, 36 are female, 4 had suspected seizure but without diagnosis, 9 were diagnosed with epilepsy, and 19 have history of febrile convulsion but ceased medication after the episodes.

|  | **Mean ± SD*** | | **Median** | |
| --- | --- | --- | --- | --- |
| **Intelligence quotient (IQ)** | | |  | |
| Performance IQ | 97.0 ± 20.4 | | 99.0 | |
| Verbal IQ | 95.4 ± 23.4 | | 99.0 | |
| Full-scale IQ | 95.1 ± 22.6 | | 99.0 | |
| **Social Responsiveness Scale (SRS)** | 88.8 ± 25.8 | | 87.0 | |
| **Swanson, Nolan and Pelham Questionnaire (SNAP)** | 26.7 ± 11.5 | | 25.0 | |
| **Autism Diagnostic Interview-Revised (ADI-R)** | **past** | **current** | **past** | **current** |
| Qualitative abnormalities in reciprocal social interaction | 20.4 ± 6.1 | 12.4 ± 5.7 | 21 | 12 |
| Qualitative abnormalities in verbal and nonverbal communication | 15.0 ± 4.4 | 9.8 ± 4.2 | 15 | 10 |
| Qualitative abnormalities in nonverbal communication | 8.2 ± 3.3 | 4.8 ± 3.5 | 8 | 4 |
| Restricted, repetitive, and stereotyped patterns of behaviour | 6.9 ± 2.5 | 5.4 ± 2.5 | 7 | 5 |
| Abnormality of development evident at or before 36 months | 3.4 ± 1.5 | - | 4 | - |

*Standard deviation (SD) calculated as the arguments are a sample of the population.

**Supplementary Table 2. The clinical data of the ASD patients in which the CNVs were located at the “hot spots”.** (Epilepsy 0 = never, 1 = suspected seizure but without diagnosis, 2 = diagnosed with epilepsy, 7 = history of febrile convulsion but ceased medication after episodes)

| **ID** | | **U-1067** | **U-1199** | **U-1344** | **U-1459** | **U-1638** | **U-1807** | **U-1902** | **U-1957** | **U-1994** | **U-2158** | **U-2233** | **U-2239** | **U-801** | **U-830** |
| --- | --- | --- | --- | --- | --- | --- | --- | --- | --- | --- | --- | --- | --- | --- | --- |
| **Sex** | | male | male | male | male | n/a | male | male | male | male | male | male | male | male | male |
| **History of Epilepsy*** | | 0 | 0 | 0 | 0 | n/a | 0 | 0 | 0 | 0 | 0 | 7 | 0 | 0 | 2 |
| **Intelligence Quotient (IQ)** | |  |  |  |  |  |  |  |  |  |  |  |  |  |  |
| Performance IQ | | 81 | n/a | 78 | n/a | n/a | 70 | n/a | n/a | 94 | 112 | 69 | 72 | 112 | 48 |
| Verbal IQ | | 75 | n/a | 91 | n/a | n/a | 74 | n/a | n/a | 116 | 95 | 89 | 57 | 120 | 70 |
| Full-scaled IQ | | 75 | n/a | 83 | n/a | n/a | 69 | n/a | n/a | 106 | 103 | 79 | 62 | 117 | 55 |
| **Social Responsiveness Scale** | | 28 | 131 | 104 | 119 | n/a | 117 | 117 | 129 | 129 | 71 | 101 | 75 | 108 | 92 |
| **Swanson, Nolan and Pelham Questionnaire** | | 5 | 41 | 25 | 35 | n/a | 22 | 50 | 41 | 42 | 28 | 38 | 23 | 18 | 23 |
| **Autism Diagnostic Interview-Revised** | |  |  |  |  |  |  |  |  |  |  |  |  |  |  |
| Qualitative abnormalities in reciprocal social interaction | *past* | 10 | 23 | 19 | 27 | n/a | 25 | 25 | 29 | 11 | 8 | 24 | 21 | 27 | 23 |
|  | *current* | 4 | 18 | 10 | 24 | n/a | 8 | 18 | 27 | 7 | 0 | 22 | 13 | 11 | 11 |
| Qualitative abnormalities in verbal and nonverbal communication | *past* | 14 | n/a | 13 | n/a | n/a | 20 | 21 | n/a | 9 | 10 | 22 | 16 | 12 | 20 |
|  | *current* | 6 | n/a | 7 | n/a | n/a | 9 | 19 | n/a | 2 | 2 | 17 | 11 | 6 | 8 |
| Qualitative abnormalities in nonverbal communication | *past* | 8 | 12 | 8 | 14 | n/a | 11 | 12 | 12 | 5 | 5 | 11 | 8 | 11 | 10 |
|  | *current* | 2 | 7 | 3 | 12 | n/a | 2 | 10 | 11 | 0 | 0 | 8 | 4 | 5 | 1 |
| Restricted, repetitive, and stereotyped patterns of behaviour | *past* | 9 | 6 | 8 | 7 | n/a | 11 | 7 | 8 | 2 | 6 | 9 | 7 | 5 | 10 |
|  | *current* | 7 | 6 | 5 | 5 | n/a | 10 | 6 | 8 | 2 | 5 | 8 | 7 | 3 | 9 |
| Abnormality of development evident at or before 36 months | *past* | 4 | 5 | 4 | 5 | n/a | 4 | 4 | 5 | 2 | 2 | 3 | 4 | 3 | 5 |

*The patient’s epilepsy history was recorded as 0 = never, 1 = suspected seizure but without diagnosis, 2 = diagnosed with epilepsy, and 7 = history of febrile convulsion but ceased medication after episodes.

**Supplementary Table 3 The clinical Data of the ASD patients in which the other rare pathogenic CNVs were identified.**

| **(a) Sex, history of epilepsy, intelligence quotient (IF), total score of social responsiveness scale (SRS), and total score of Swanson, Nolan and Pelham Questionnaire (SNAP-IV).** | | | | | | | |
| --- | --- | --- | --- | --- | --- | --- | --- |
| **ID** | **Sex** | **Epilepsy*** | **Intelligence Quotient (IQ)** | | | **SRS**  **(autistic-like social deficits)** | **SNAP-IV**  **Symptoms of inattention, hyperactivity, and oppositional** |
|  |  |  | **Performance IQ** | **Verbal IQ** | **Full-scale IQ** |  |  |
| **U-1130** | male | 0 | 75 | 49 | 61 | 102 | 25 |
| **U-1160** | male | 7 | 109 | 122 | 118 | n/a | n/a |
| **U-1230** | male | 0 | 97 | 73 | 81 | 111 | 20 |
| **U-1255** | male | 0 | n/a | n/a | n/a | 137 | 25 |
| **U-1269** | male | 0 | 100 | 107 | 105 | 84 | 6 |
| **U-1340** | male | 0 | 102 | 87 | 94 | 84 | 22 |
| **U-1385** | male | 7 | 106 | 120 | 114 | 85 | 25 |
| **U-1414** | male | 0 | n/a | n/a | n/a | 102 | 45 |
| **U-1428** | female | 0 | 72 | 73 | 69 | 91 | 22 |
| **U-1452** | male | 0 | 107 | 102 | 105 | 104 | 29 |
| **U-1511** | male | 0 | n/a | n/a | n/a | 89 | 43 |
| **U-1519** | male | 0 | 94 | 105 | 100 | 83 | 30 |
| **U-1535** | male | 0 | 77 | 93 | 83 | 45 | 32 |
| **U-1578** | male | 0 | 101 | 105 | 104 | 41 | 18 |
| **U-1626** | male | 7 | 120 | 111 | 116 | 87 | 17 |
| **U-1691** | male | 0 | 97 | 99 | 97 | 51 | 42 |
| **U-1726** | n/a | n/a | n/a | n/a | n/a | n/a | n/a |
| **U-1753** | male | 7 | 100 | 105 | 103 | 59 | 25 |
| **U-1850** | male | 0 | 105 | 114 | 111 | 95 | 41 |
| **U-1924** | male | 0 | n/a | n/a | 99 | 94 | 25 |
| **U-1957** | male | 0 | n/a | n/a | n/a | 129 | 41 |
| **U-1967** | male | 0 | 102 | 88 | 93 | 82 | 49 |
| **U-1999** | male | 0 | 95 | 88 | 90 | 79 | 30 |
| **U-2015** | male | 0 | 109 | 105 | 107 | 93 | 31 |
| **U-2058** | male | 0 | 98 | 114 | 107 | 120 | 48 |
| **U-2075** | male | 0 | n/a | n/a | n/a | 82 | 22 |
| **U-212** | n/a | n/a | n/a | n/a | n/a | n/a | n/a |
| **U-2131** | male | 0 | 118 | 113 | 116 | 96 | 15 |
| **U-215** | male | 0 | n/a | n/a | n/a | 79 | n/a |
| **U-2170** | male | 0 | 97 | 95 | 95 | 45 | 9 |
| **U-2200** | male | 0 | 116 | 84 | 97 | 108 | 47 |
| **U-273** | n/a | n/a | n/a | n/a | n/a | n/a | n/a |
| **U-2829** | male | 0 | 113 | 90 | 100 | 79 | 25 |
| **U-363** | male | 0 | 105 | 104 | 105 | 96 | 30 |
| **U-480** | male | 0 | n/a | n/a | n/a | 79 | n/a |
| **U-717** | male | 0 | 109 | 109 | 110 | 132 | 21 |
| **U-728** | male | 0 | 107 | 109 | 108 | 107 | 33 |
| **U-754** | male | 0 | 74 | 89 | 80 | 48 | 3 |
| **U-866** | male | 0 | 88 | 46 | 66 | 45 | 3 |
| **U-890** | male | 0 | n/a | n/a | n/a | 78 | n/a |
| **U-919** | male | 0 | 105 | 98 | 101 | 53 | 30 |
| **U-925** | male | 0 | 95 | 109 | 103 | 104 | 29 |
| **U-985** | male | 0 | 118 | 107 | 112 | 74 | 16 |

*The patient’s epilepsy history was recorded as 0 = never, 1 = suspected seizure but without diagnosis, 2 = diagnosed with epilepsy, and 7 = history of febrile convulsion but ceased medication after episodes.

| **(b) Autism Diagnostic Interview-Revised (ADI-R) Scores.** | | | | | | | | | |
| --- | --- | --- | --- | --- | --- | --- | --- | --- | --- |
|  | **Qualitative abnormalities in reciprocal social interaction** | | **Qualitative abnormalities in verbal and nonverbal communication** | | **Qualitative abnormalities in nonverbal communication** | | **Restricted, repetitive, and stereotyped patterns of behaviour** | | **Abnormality of development evident at or before 36 months** |
| **ID** | past | current | past | current | past | current | past | current | past |
| **U-1130** | 23 | 9 | 15 | 10 | 7 | 4 | 7 | 3 | 5 |
| **U-1160** | 22 | 20 | 17 | 15 | 12 | 12 | 5 | 5 | 5 |
| **U-1230** | 26 | 16 | 17 | 12 | 8 | 6 | 10 | 9 | 5 |
| **U-1255** | 28 | 20 | 14 | 9 | 7 | 2 | 12 | 11 | 2 |
| **U-1269** | 24 | 12 | 20 | 11 | 12 | 6 | 10 | 2 | 5 |
| **U-1340** | 27 | 18 | 14 | 8 | 10 | 4 | 5 | 3 | 2 |
| **U-1385** | 13 | 7 | 9 | 7 | 5 | 5 | 5 | 5 | 1 |
| **U-1414** | 23 | 15 | 23 | 19 | 12 | 9 | 7 | 6 | 5 |
| **U-1428** | 13 | 7 | 13 | 10 | 8 | 5 | 1 | 0 | 2 |
| **U-1452** | 27 | 9 | 12 | 7 | 8 | 3 | 7 | 2 | 4 |
| **U-1511** | 29 | 27 | n/a | n/a | 14 | 12 | 6 | 6 | 5 |
| **U-1519** | 8 | 5 | 9 | 9 | 4 | 4 | 1 | 1 | 0 |
| **U-1535** | 8 | 2 | 7 | 2 | 3 | 1 | 0 | 0 | 0 |
| **U-1578** | 14 | 6 | 8 | 6 | 2 | 2 | 6 | 6 | 4 |
| **U-1626** | 25 | 13 | 16 | 7 | 8 | 2 | 7 | 6 | 3 |
| **U-1691** | 18 | 7 | 10 | 5 | 6 | 2 | 1 | 1 | 5 |
| **U-1726** | n/a | n/a | n/a | n/a | n/a | n/a | n/a | n/a | n/a |
| **U-1753** | 5 | 4 | 10 | 8 | 4 | 4 | 4 | 3 | 0 |
| **U-1850** | 22 | 11 | 11 | 6 | 6 | 3 | 8 | 6 | 3 |
| **U-1924** | 26 | 16 | 16 | 12 | 9 | 8 | 10 | 6 | 3 |
| **U-1957** | 29 | 27 | n/a | n/a | 12 | 11 | 8 | 8 | 5 |
| **U-1967** | 25 | 20 | 16 | 11 | 7 | 6 | 10 | 8 | 5 |
| **U-1999** | 22 | 7 | 18 | 8 | 7 | 0 | 8 | 7 | 5 |
| **U-2015** | 8 | 3 | 7 | 5 | 1 | 0 | 7 | 7 | 0 |
| **U-2058** | 25 | 13 | 15 | 6 | 7 | 0 | 9 | 6 | 2 |
| **U-2075** | 28 | 17 | 18 | 12 | 10 | 4 | 10 | 8 | 5 |
| **U-212** | n/a | n/a | n/a | n/a | n/a | n/a | n/a | n/a | n/a |
| **U-2131** | 25 | 17 | 18 | 9 | 9 | 4 | 11 | 5 | 5 |
| **U-215** | 12 | 12 | 10 | 10 | 2 | 2 | 9 | 9 | 4 |
| **U-2170** | 25 | 9 | 19 | 11 | 12 | 6 | 4 | 4 | 3 |
| **U-2200** | 15 | 5 | 21 | 3 | 11 | 1 | 12 | 12 | 5 |
| **U-273** | n/a | n/a | n/a | n/a | n/a | n/a | n/a | n/a | n/a |
| **U-2829** | 21 | 9 | 16 | 7 | 7 | 0 | 6 | 6 | 4 |
| **U-363** | 13 | 7 | 7 | 4 | 0 | 0 | 5 | 0 | 2 |
| **U-480** | 26 | 25 | 14 | 14 | 6 | 6 | 8 | 3 | 5 |
| **U-717** | 25 | 14 | 23 | 7 | 14 | 0 | 9 | 6 | 5 |
| **U-728** | 23 | 13 | 13 | 8 | 6 | 2 | 1 | 1 | 1 |
| **U-754** | 11 | 11 | 8 | 3 | 4 | 0 | 6 | 4 | 2 |
| **U-866** | 26 | 7 | 15 | 7 | 6 | 0 | 3 | 3 | 5 |
| **U-890** | 18 | 17 | 22 | 22 | 14 | 14 | 9 | 5 | 4 |
| **U-919** | 21 | 19 | 18 | 17 | 8 | 7 | 8 | 8 | 4 |
| **U-925** | 28 | 13 | 16 | 10 | 8 | 3 | 7 | 5 | 2 |
| **U-985** | 19 | 7 | 13 | 5 | 7 | 1 | 7 | 4 | 3 |

**Supplementary Table 4. The clinical Data of ASD patients in which two hits of CNV were identified.**

| **ID** |  | **U-1255** | **U-1414** | **U-1753** | **U-1999** | **U-2075** |
| --- | --- | --- | --- | --- | --- | --- |
| **Sex** |  | 1 | 1 | 1 | 1 | 1 |
| **History of Epilepsy*** |  | 0 | 0 | 7 | 0 | 0 |
| **Intelligence Quotient (IQ)** |  |  |  |  |  |  |
| Performance IQ |  | n/a | n/a | 100 | 95 | n/a |
| Verbal IQ |  | n/a | n/a | 105 | 88 | n/a |
| Full-scaled IQ |  | n/a | n/a | 103 | 90 | n/a |
| **Social Responsiveness Scale** |  | 137 | 102 | 59 | 79 | 82 |
| **Swanson, Nolan and Pelham Questionnaire** |  | 25 | 45 | 25 | 30 | 22 |
| **Autism Diagnostic Interview-Revised** |  |  |  |  |  |  |
| Qualitative abnormalities in reciprocal social interaction | *past* | 28 | 23 | 5 | 22 | 28 |
|  | *current* | 20 | 15 | 4 | 7 | 17 |
| Qualitative abnormalities in verbal and nonverbal communication | *past* | 14 | 23 | 10 | 18 | 18 |
|  | *current* | 9 | 19 | 8 | 8 | 12 |
| Qualitative abnormalities in nonverbal communication | *past* | 14 | 23 | 10 | 18 | 18 |
|  | *current* | 2 | 9 | 4 | 0 | 4 |
| Restricted, repetitive, and stereotyped patterns of behaviour | *past* | 12 | 7 | 4 | 8 | 10 |
|  | *current* | 11 | 6 | 3 | 7 | 8 |
| Abnormality of development evident at or before 36 months (D) | *past* | 2 | 5 | 0 | 5 | 5 |

*The patient’s epilepsy history was recorded as 0 = never, 1 = suspected seizure but without diagnosis, 2 = diagnosed with epilepsy, and 7 = history of febrile convulsion but ceased medication after episodes.
